# Immune Response Visualized In Vivo by [18F]-FDG PET/CT after COVID-19 Vaccine

**DOI:** 10.3390/diagnostics11040676

**Published:** 2021-04-09

**Authors:** Romain-David Seban, Laurence Champion, Nicolas Deleval, Capucine Richard, Claire Provost

**Affiliations:** 1 Department of Nuclear Medicine and Endocrine Oncology, Institut Curie, 92210 Saint-Cloud, France; laurence.champion@curie.fr (L.C.); nicolas.deleval@curie.fr (N.D.); capucine.richard@curie.fr (C.R.); 2 Laboratoire d’Imagerie Translationnelle en Oncologie, Inserm, Institut Curie, 91401 Orsay, France; claire.provost@curie.fr; 3 Department of Pharmacology, Institut Curie, 92210 Saint-Cloud, France

**Keywords:** [18F]-FDG PET/CT, COVID-19 vaccine, spleen, lymph nodes, immune response

## Abstract

Worldwide deployment of COVID-19 vaccines is in progress. Recent immune activation following vaccination can sometimes be seen in fluorine-18-fluorodeoxyglucose positron emission tomography/computed tomography ([18F]-FDG PET/CT). As previously evidenced, FDG-avid axillary lymph node(s) are common in patients receiving vaccines against SARS-CoV-2, influenza virus, or human papillomavirus, and reflect a regional immune response. In addition, these findings may also be accompanied by an increased spleen glucose metabolism after the COVID-19 vaccine, which captures a systemic immune response. Hence, we provide here a clinical example demonstrating that immune response could be associated with increased glucose metabolism in lymphoid organs such as lymph nodes and the spleen, which are critical modulators of T cell immunity. We believe that it is of paramount importance that nuclear physicians should be able to recognize clinical and imaging features of such immune responses upon vaccination for COVID-19 and beyond.

We report the case of a 67-year-old woman who had been treated for an early stage left invasive breast carcinoma with tumorectomy. Given the positive sentinel lymph node, she underwent a staging [18F]-FDG PET/CT twenty days after surgery, which showed a diffusely high spleen glucose metabolism associated with hypermetabolic right axillary lymph nodes (Figure 1). Five days before imaging, she had been administered her first dose of the COVID-19 viral vector vaccine (AZD1222) in the right upper arm without any early side effects.

Upon questioning, the patient had no history of treatment or disease that could potentially influence metabolic activity in lymphoid tissues such as corticosteroids or G(M)-CSF [2] received over the last six months, history of chronic inflammatory [3], auto-immune, or hematologic [4] or infectious disease [5]. We thus assumed that the high glucose metabolism within the right axillary lymph nodes and spleen were presumably attributable to a regional and systemic immune response, respectively.

While hypermetabolic axillary lymph nodes have been widely described upon COVID-19 vaccination, as well as with messenger RNA [6] or vector vaccines [7], the increased uptake within the whole spleen seems more scarce. Steinberg et al. recently reported the case of a 65-year-old woman who had her first dose of the mRNA-1273 COVID-19 vaccine and who developed a systemic inflammatory response syndrome (SIRS) within 1 day of the injection [8]. The symptoms finally resolved 3 days after the vaccination, and the [18F]-FDG PET/CT performed 2 days later revealed a diffusely high spleen glucose metabolism associated with hypermetabolic right axillary lymph nodes (vaccination in the right deltoid). Therefore, our clinical presentation differs in two respects: on the one hand, our patient received a viral vector vaccine, and on the other hand, she remained asymptomatic in the days following the vaccination.

Overall, this brief report suggests that nuclear physicians should be aware of the clinical and imaging patterns of the immune response to vaccination, especially in the context of the current COVID-19 pandemic.

In conclusion, these recent findings open up new prospects for the future beyond COVID-19, notably with the emergence of the cancer vaccine in immuno-oncology [9]. Extending that thought, we might expect to visualize anti-tumor immune responses in vivo with [18F]-FDG PET/CT. Nevertheless, further studies are warranted to investigate and clarify this potential new indication for [18F]-FDG PET/CT.

## Figures and Tables

**Figure 1:**
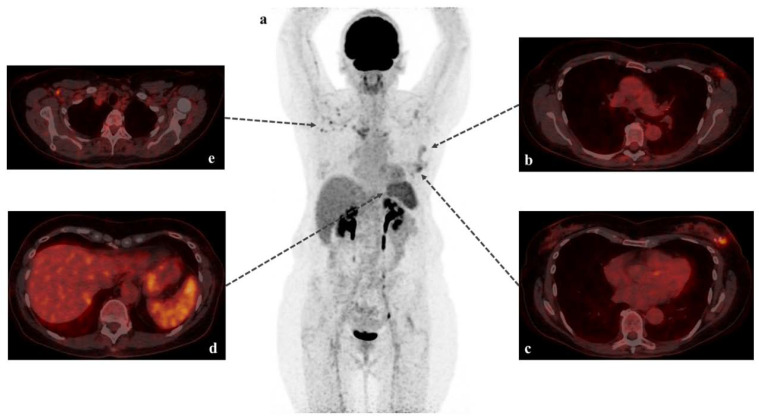
[18F]-FDG PET/CT scans with MIP (maximum intensity projection: (**a**) and fused axial images (**b**–**e**). Staging [18F]-FDG PET/CT revealed postoperative aspects in the left breast and ipsilateral axilla (**b**,**c**). Interestingly, we also found a diffusely high spleen glucose metabolism (**d**): maximum and mean standardized uptake value of 5.5 and 4.3, respectively), which was higher than the normal liver uptake (maximum and mean standardized uptake value of 4.4 and 3.2, respectively) frequently used as an internal reference organ to assess the significance of FDG uptake in pathologic processes. These findings were associated with hypermetabolic right axillary lymph nodes (**e**): maximum and mean standardized uptake values of 5.6 and 3.0, respectively). No pathological uptake of FDG was detected within the subcutaneous soft tissues of the right deltoid, corresponding to the injection site of the viral vector vaccine (AZD1222) [1].

## Data Availability

The data presented in this study are available on request from thecorresponding author.
